# Are maternal serum subfatin levels altered in women with one abnormal glucose tolerance test value?

**DOI:** 10.1590/1806-9282.20231111

**Published:** 2024-08-16

**Authors:** Yıldız Akdas Reis, Fahri Burcin Firatligil, Alperen Aksan, Caner Kose, Harun Egemen Tolunay, Yaprak Ustun

**Affiliations:** 1 University of Health Sciences Turkey, Ankara Etlik Zubeyde Hanım Women's Health and Research Center Turkey, Department of Obstetrics and Gynecology - Ankara, Turkey.

**Keywords:** Cholesterol, Gestational diabetes mellitus, Glucose tolerance test, Subfatin, Triglyceride

## Abstract

**BACKGROUND:**

Subfatin, a newly discovered adipokine, plays a pivotal role in the regulation of glucose metabolism. The relationship between gestational diabetes mellitus and maternal dyslipidemia is well-documented.

**AIMS:**

This study aims to assess serum subfatin levels and the triglyceride/high-density lipoprotein cholesterol ratio in women with one abnormal glucose tolerance test value and those with gestational diabetes mellitus.

**METHODS:**

In this case-control study, 105 pregnant women were categorized into three groups: women with normal 3-h oral glucose tolerance test results (n=35), women with one abnormal 3-h oral glucose tolerance test result (n=35), and women diagnosed with gestational diabetes mellitus (n=35). Serum subfatin levels were measured using human enzyme-linked immunosorbent assay kits.

**RESULTS:**

Serum subfatin levels were significantly lower in the gestational diabetes mellitus group (0.94±0.15 ng/mL) compared to the normal oral glucose tolerance test group (1.48±0.55 ng/mL) and the group with one abnormal oral glucose tolerance test result (1.50±0.59 ng/mL). The triglyceride/high-density lipoprotein cholesterol ratio was also lower in the healthy control group than in the gestational diabetes mellitus and one abnormal oral glucose tolerance test result groups.

**CONCLUSION:**

Serum subfatin levels in women with one abnormal abnormal glucose tolerance test value are compared to those in the control group, while the triglyceride/high-density lipoprotein cholesterol ratio is significantly altered in women with one abnormal abnormal glucose tolerance test value when compared to the control group.

## INTRODUCTION

Gestational diabetes mellitus (GDM) is described as insulin resistance that emerges or is first identified during pregnancy^1^. As the prevalence of GDM continues to rise, understanding the mechanisms underlying glucose regulation and insulin resistance during pregnancy becomes increasingly crucial. Various studies have proposed a relationship between adipokines and GDM^2-4^. Unlike most adipokines identified in obesity models, subfatin was first described in the PGC-1α4 transgenic mice model^5^. Subfatin, an adipokine predominantly produced by adipose tissue, is crucial in regulating insulin sensitivity through the peroxisome proliferator-activated receptor-gamma (PPAR-γ) pathway^2-6^. Besides, subfatin is particularly intriguing due to its dual role in enhancing energy expenditure through the browning of white adipose tissue and its potential impact on insulin sensitivity^7^. However, findings on the relationship between subfatin levels and GDM show discrepancies. For instance, Yavuzkir et al. discovered that subfatin levels were significantly elevated in mothers with GDM compared to those with normal pregnancies^2^. Conversely, subfatin levels have been associated with a negative correlation with serum glucose levels and an exacerbation of glucose tolerance test (GTT) outcomes^8,9^. A single abnormal value on an oral glucose tolerance test (OGTT) is considered a pathological indicator. Patients with a single abnormal test result exhibited no difference from those diagnosed with GDM in terms of fasting insulin levels and insulin resistance^10^. This study also aims to explore if patients with a single abnormal test result exhibit altered subfatin levels.

The triglyceride (TG) to high-density lipoprotein cholesterol (HDL-C) ratio has been positively linked to insulin resistance^11^. Significant disparities in the TG/HDL-C ratio have been observed between women with and without GDM^12^. For instance, lower HDL-C^13^ and the TG/HDL-C ratio^14^ are indicators of insulin resistance. Since insulin resistance is a key underlying factor of GDM, various lipid ratios have been utilized to assess GDM risk^15^. The early diagnosis of GDM is crucial, yet there is a lack of comprehensive information on universal or case-specific screening tests for GDM. Coupled with alterations in lipid metabolism in GDM patients and the potential pathophysiology of the disease, there is a pressing need for additional research like this study. This study aims to examine serum subfatin levels and the TG/HDL-C ratio in women with abnormal GTTs (AGTT) and GDM, and its correlation with AGTT and its broader implications for insulin resistance in GDM. By exploring the function of subfatin, this research seeks to uncover novel insights into the metabolic adjustments during pregnancy that could influence the development and management of GDM.

## METHODS

This case-control study was conducted at the Obstetrics, Gynecology, and Perinatology Clinics of Etlik Zubeyde Hanim Women's Health Education and Training Hospital between March 2021 and October 2021. Approval was obtained from the local ethics committee on December 30, 2020 (Approval No: 2020/175).

### Inclusion-exclusion criteria

In diagnosing GDM, we opted for the National Institute for Health and Care Excellence (NICE) guidelines rather than the International Association of Diabetes in Pregnancy Study Groups (IADPSG) recommendations^16^. This decision was informed by this study, which indicates that the NICE guidelines provide a more favorable cost-benefit ratio in terms of diagnostic efficacy and healthcare resource allocation^16^. Details of this study and the rationale for our choice are briefly outlined in this section to assist other researchers in understanding the contextual factors influencing the selection of diagnostic criteria.

The study included singleton pregnancies of women aged 18-39 years within 24-28 gestational weeks. Exclusion criteria encompassed any of the following: a history of macrosomia (birth weight >4,000 g) or stillbirth, previous GDM, congenital malformations or chromosomal abnormalities in the fetus, fetal death, multiple pregnancies, maternal polycystic ovary syndrome, pregestational diabetes mellitus (DM) or a first-degree relative with DM, pregnancy-induced hypertension, chronic maternal diseases (such as chronic hypertension, dyslipidemia, renal failure, or diseases of the thyroid, liver, lung, or heart, and malignancy), use of drugs affecting lipid or glucose metabolism, and alcohol consumption.

### Study design

GDM screening was performed using a 50 g glucose challenge test (GCT) between 24 and 28 gestational weeks. Pregnant women with GCT results above 140 mg/dL underwent a 3-h OGTT and were categorized into three groups, each comprising 35 participants: women with normal OGTT results (Group 1), women with one abnormal OGTT value (Group 2), and women diagnosed with GDM (Group 3).

### Collection and storage of biological samples

Blood samples were collected from participants between 24 and 28 gestational weeks to measure serum subfatin, insulin, selected serum lipid profiles (TG, LDL, VLDL, HDL-C), and glucose levels. About 8 mL of blood, drawn using sterile syringes, was centrifuged at 3,000 × g for 5 min. The plasma was then aliquoted into Eppendorf tubes and stored at −80°C until analysis.

### Laboratory analysis of biological samples

Serum subfatin levels were measured using human enzyme-linked immunosorbent assay (ELISA) kits (Catalog No. E3941Hu, HEALES MB-530, Shanghai, China). The standard curve for subfatin ranged from 0.05 to 15 ng/mL, with a sensitivity of 0.023 ng/mL. Lipid profiles and blood glucose levels were analyzed using the Advia 2400 Clinical Chemistry System (Siemens, Tarrytown, NY, USA).

We compared the fasting plasma glucose levels, fasting serum insulin levels, selected serum lipid metabolism parameters (TC, LDL-C, HDL-C, VLDL-C, and TG), and specifically serum subfatin levels obtained between the 24th and 28th weeks of pregnancy.

### Statistical analyses

Data were analyzed using the SPSS software, version 21.0 (IBM Corporation, Armonk, NY, USA)^17^. Power analysis was conducted with G*Power 3.1, suggesting a sample size of approximately 70 cases and 35 controls to detect the association with 95% power at a 0.05 alpha level^18^. Group comparisons and categorical variables were analyzed using the chi-square and ANOVA tests. The Kruskal-Wallis test was applied for comparisons among the three groups, followed by the Mann-Whitney U-test for post-hoc analysis upon detecting significant differences. The association strength between parameters was assessed using Spearman's rank correlation coefficient, with a p-value of 0.05 deemed significant.

## RESULTS

The demographic characteristics of the study groups are presented in Table 1. There were no statistically significant differences across the groups in terms of maternal age, body mass index (BMI), smoking habits, number of pregnancies (gravida), number of births (parity), and gestational age at the time of the OGTT.

**Table 1:** Demographic characteristics of the study groups.

		Normal 3-h OGTT (n=35)	One abnormal 3-h OGTT (n=35)	GDM (n=35)	Chi-square	p-value
		n (%)	n (%)	n (%)		
Age (years)	<31	18 (51.4)	16 (45.7)	15 (42.9)	1.163	0.884
	31-35	9 (25.7)	9 (25.7)	8 (22.9)		
	>35	8 (22.9)	10 (28.6)	12 (34.3)		
BMI (kg/m^2^)	18.5-24.9	9 (25.7)	4 (11.4)	5 (14.3)	8.068	0.089
	25-29.9	15 (42.9)	12 (34.3)	20 (57.1)		
	30-34.9	11 (31.4)	19 (54.3)	10 (28.6)		
Smoking status	Smoker	3 (8.6)	5 (14.3)	5 (14.3)	0.702	0.704
	Nonsmoker	32 (91.4)	30 (85.7)	30 (85.7)		
		Mean±SD	Mean±SD	Mean±SD	F	p-value
Gravida		2.94±1.39	2.69±1.37	3.06±1.57	2.744	0.840
Parity		1.48±0.95	1.11±0.83	1.40±1.06	6.293	0.391
Gestational age at OGTT (weeks)		26.22±1.42	26.40±1.48	26.40±1.38	17.036	0.058

Significant differences were observed in fasting plasma glucose and serum TG levels among the groups, with p-values <0.05. The TG/HDL-C ratio varied widely, ranging from 0.82 to 9.11, with an average ratio of 3.23±1.52. This ratio also showed significant variation among the groups, as indicated by a p-value <0.05. Serum subfatin levels were found to be between 0.71 and 3.82 ng/mL, averaging 1.31±0.54 ng/mL. The difference in serum subfatin levels was statistically significant across the groups, with a p-value <0.05 (Table 2).

**Table 2:** Comparison of biochemical parameters among the study groups.

	Normal 3-h OGTT (n=35)	One abnormal 3-h OGTT (n=35)	GDM (n=35)	f	p-value
	Mean±SD	Mean±SD	Mean±SD		
FBG (mg/dL)	78.11±6.62	82.22±21.13	91.31±14.56	6.693	0.002
Fasting insulin (mIU/mL)	9.95±7.57	15.00±17.10	15.73±14.46	1.861	0.161
TC (mg/dL)	252.40±46.74	244.92±48.53	245.76±56.35	0.229	0.796
LDL-C (mg/dL)	145.39±34.80	137.87±44.45	136.89±45.28	0.434	0.649
HDL-C (mg/dL)	70.01±14.79	67.96±13.20	65.49±14.57	0.886	0.415
VLDL-C (mg/dL)	37.86±11.53	39.77±14.91	45.92±16.36	2.991	0.055
TG (mg/dL)	187.11±58.81	198.69±74.58	231.14±82.09	3.471	0.035
TG/HDL-C ratio	2.80±1.06	3.13±1.61^a^	3.75±1.69^a^	3.720	0.028
				*	p-value
Serum subfatin levels (ng/mL)	1.48±0.55	1.50±0.59^b^	0.94±0.15^c^	47.561	<0.001

Data are presented as the mean ± standard deviation (SD).

FBG, fasting blood glucose

TC, total cholesterol

LDL-C, low-density lipoprotein cholesterol

HDL-C, high-density lipoprotein cholesterol

VLDL-C, very-low-density lipoprotein cholesterol

TG, triglyceride; F, value get after ANOVA test.

a p < 0.05 compared with the control group.

b p = NS compared with the control group.

c p <0.001 compared with the control group and one AGTT value group.

* Kruskal-Wallis test.

However, there was no significant correlation between serum subfatin levels and the TG/HDL-C ratio (rho=-0.096; p=0.332), suggesting that while both parameters significantly varied among the groups, they did not show a direct relationship with each other.

## DISCUSSION

To the best of our knowledge, this study is the inaugural exploration of subfatin levels and the TG/HDL-C ratio in individuals with one AGTT value. Extensive research has been conducted to uncover the mechanisms behind GDM, focusing on the role of adipokines and myokines^2,19,20^. In recent years, the study of hormones derived from adipokines and myokines has become crucial for understanding GDM's underlying mechanisms^21,22^. Subfatin, a highly expressed adipokine in subcutaneous fat, has been scrutinized for its potential involvement in GDM^2,6-9^, yielding mixed results that underscore the need for further investigation.

Wang K et al. embarked on a study to examine the relationship between meteorin-like protein (Metrnl) serum levels, blood glucose status, and insulin resistance^23^. Their findings suggest that elevated serum levels of Metrnl (including subfatin, cometin, etc.) are significantly associated with an increased risk of type 2 DM, independent of insulin resistance^23^. Similarly, Chung et al. found that Metrnl levels were significantly higher in diabetic patients compared to a healthy control group, with levels correlating with fasting plasma glucose and lipid profiles after adjusting for age and sex^24^. Contrary to Wang K et al. and Chung et al., Dadmanesh et al. reported decreased serum Metrnl levels in individuals with type 2 DM^8^. Our study revealed that serum subfatin levels were lower in the GDM group but higher in the AGTT group, with control group levels falling between these groups. Dadmanesh et al. suggested that discrepancies in subfatin levels might stem from medical treatments or ethnic differences^6^. Contrasting with Chung et al., Lee et al. found that medical treatment did not affect serum Metrnl levels after 12 weeks^25^. In our research, participants with GDM and/or AGTT were not subjected to any medical treatment apart from dietary advice at the time of diagnosis; hence, their serum subfatin levels were measured.

We also observed an increase in serum subfatin levels in the AGTT group and a decrease in the GDM group, aligning with Dadmanesh et al's findings^8^. There are limited studies on individuals with type 2 diabetes, and only one study discusses serum subfatin level changes in GDM^2,8,21-25^. This makes our study the first to report on maternal serum subfatin levels in women with GDM and one AGTT value.

Yavuzkir et al. conducted a study to assess serum subfatin levels in GDM patients^2^. Their results indicated that GDM led to increased subfatin levels, suggesting the protein's potential as a biomarker for GDM diagnosis and management^2^. Our findings show that serum subfatin levels increase with one AGTT value and decrease in GDM cases, statistically.

Furthermore, our study found a significant difference in TG/HDL-C ratios among groups, with non-GDM or one AGTT value individuals exhibiting lower TG/HDL-C ratios than those with GDM or one AGTT value. This is consistent with other studies that affirm the link between hyperlipidemia and GDM^12,26,27^. However, some research contradicts this, pointing to the influence of diet, exercise, lifestyle, and ethnicity on lipid profiles^12,15,26,27^.

In this study, we restricted our women participants’ age to 39 years and younger. This decision was based on data suggesting that metabolic and hormonal profiles can differ significantly in women aged over 40 years, potentially confounding the effects of subfatin levels on glucose tolerance and GDM outcomes. Research indicates that age-related hormonal changes, especially around the perimenopausal period, can significantly alter glucose metabolism and insulin sensitivity, which might mask the specific effects of subfatin we aimed to investigate. Furthermore, age-related increases in the prevalence of comorbid conditions such as cardiovascular disease and type 2 diabetes could introduce additional variability, complicating the interpretation of our findings. Thus, focusing on a younger cohort allows for a more controlled analysis of subfatin's role in the early stages of metabolic dysregulation typically observed in pregnancy and pre-diabetic states^28^.

This study marks a significant contribution to GDM research by pioneering the investigation of serum subfatin levels and the TG/HDL-C ratio in individuals with one abnormal AGTT value, offering valuable insights into metabolic changes associated with GDM. The observed increase in subfatin levels in subjects with abnormal AGTT values suggests a potential compensatory mechanism that enhances insulin sensitivity and glucose regulation. This aligns with subfatin's known function in promoting the browning of adipose tissue, a process that not only increases energy expenditure but also improves insulin action. The relationship between subfatin and insulin resistance is critical, especially given that its upregulation in response to metabolic stress (such as exercise and cold exposure) has been shown to improve glucose uptake and metabolic health^29^. However, the dynamics of subfatin expression and its impact on insulin sensitivity during pregnancy remain complex. Its comprehensive approach, which builds on existing contradictory findings regarding subfatin levels, and its acknowledgment of potential confounders, such as medical treatment and ethnic differences, highlight its strengths. However, the study faces limitations due to its small sample size, which affects the statistical power and generalizability of its findings. Additionally, its cross-sectional design limits the ability to establish causality or observe longitudinal changes, and it does not fully address other potential confounding factors, such as lifestyle and socioeconomic status, which could influence the outcomes. Furthermore, the study's focus on a specific population may limit its applicability to wider demographic groups, and a lack of in-depth analysis into the underlying biological mechanisms connecting subfatin and lipid metabolism to GDM calls for further research. Addressing these weaknesses through larger, more diverse, and longitudinal studies could enhance the robustness and applicability of the findings, providing clearer insights into the pathophysiological pathways involved in GDM.

## CONCLUSION

The mean serum subfatin levels in women with GDM were lower than those in women with one AGTT value and healthy control groups; women with one AGTT value had the highest serum subfatin level in the present study. Also, TG/HDL-C ratio was lower in the healthy control group. Larger studies are required to clarify the relationship between subfatin and GDM and one AGTT value.

## ETHICAL APPROVAL

All procedures performed in studies involving human participants were in accordance with the ethical standards of the institutional research committee at which the studies were conducted (Etlik Zubeyde Hanim Women's Health Education and Training Hospital; December 30, 2020; no: 2020/175) and with the 2013 Helsinki Declaration and its later amendments or comparable ethical standards.

## INFORMED CONSENT

Informed consent was obtained from all individual participants included in the study.

## References

[1] Rasmussen L, Poulsen CW, Kampmann U, Smedegaard SB, Ovesen PG, Fuglsang J (2020). Diet and healthy lifestyle in the management of gestational diabetes mellitus. Nutrients.

[2] Yavuzkir S, Ugur K, Deniz R, Ustebay DU, Mirzaoglu M, Yardim M (2020). Maternal and umbilical cord blood subfatin and spexin levels in patients with gestational diabetes mellitus. Peptides.

[3] Radzicka S, Pietryga M, Iciek R, Brązert J (2018). The role of visfatin in pathogenesis of gestational diabetes (GDM). Ginekol Pol.

[4] Siddiqui K, George TP, Nawaz SS, Shehata N, El-Sayed AA, Khanam L (2018). Serum adipokines (adiponectin and resistin) correlation in developing gestational diabetes mellitus: pilot study. Gynecol Endocrinol.

[5] Rao RR, Long JZ, White JP, Svensson KJ, Lou J, Lokurkar I (2014). Meteorin-like is a hormone that regulates immune-adipose interactions to increase beige fat thermogenesis. Cell.

[6] Li ZY, Song J, Zheng SL, Fan MB, Guan YF, Qu Y (2015). Adipocyte metrnl antagonizes insulin resistance through PPARγ signaling. Diabetes.

[7] Şekerci G, Erden Y, Tekin S (2022). Effects of meteorin-like hormone on endocrine function of hypothalamo-hypophysial system and peripheral uncoupling proteins in rats. Mol Biol Rep.

[8] Dadmanesh M, Aghajani H, Fadaei R, Ghorban K (2018). Lower serum levels of Meteorin-like/Subfatin in patients with coronary artery disease and type 2 diabetes mellitus are negatively associated with insulin resistance and inflammatory cytokines. PLoS One.

[9] Fadaei R, Dadmanesh M, Moradi N, Ahmadi R, Shokoohi Nahrkhalaji A, Aghajani H (2020). Serum levels of subfatin in patients with type 2 diabetes mellitus and its association with vascular adhesion molecules. Arch Physiol Biochem.

[10] Ergin T, Lembet A, Duran H, Kuscu E, Bagis T, Saygili E (2002). Does insulin secretion in patients with one abnormal glucose tolerance test value mimic gestational diabetes mellitus?. Am J Obstet Gynecol.

[11] Gong R, Luo G, Wang M, Ma L, Sun S, Wei X (2021). Associations between TG/HDL ratio and insulin resistance in the US population: a cross-sectional study. Endocr Connect.

[12] Barat S, Ghanbarpour A, Bouzari Z, Batebi Z (2018). Triglyceride to HDL cholesterol ratio and risk for gestational diabetes and birth of a large-for-gestational-age newborn. Caspian J Intern Med.

[13] Neboh EE, Emeh JK, Aniebue UU, Ikekpeazu EJ, Maduka IC, Ezeugwu FO (2012). Relationship between lipid and lipoprotein metabolism in trimesters of pregnancy in Nigerian women: is pregnancy a risk factor?. J Nat Sci Biol Med.

[14] McLaughlin T, Abbasi F, Cheal K, Chu J, Lamendola C, Reaven G (2003). Use of metabolic markers to identify overweight individuals who are insulin resistant. Ann Intern Med.

[15] Wang D, Xu S, Chen H, Zhong L, Wang Z (2015). The associations between triglyceride to high-density lipoprotein cholesterol ratios and the risks of gestational diabetes mellitus and large-for-gestational-age infant. Clin Endocrinol (Oxf).

[16] Bhatia M, Mackillop LH, Bartlett K, Loerup L, Kenworthy Y, Levy JC (2018). Clinical implications of the NICE 2015 criteria for gestational diabetes mellitus. J Clin Med.

[17] Gouda MA (2015). Common pitfalls in reporting the use of SPSS software. Med Princ Pract.

[18] Faul F, Erdfelder E, Buchner A, Lang AG (2009). Statistical power analyses using G*Power 3.1: tests for correlation and regression analyses. Behav Res Methods.

[19] Johns EC, Denison FC, Norman JE, Reynolds RM (2018). Gestational diabetes mellitus: mechanisms, treatment, and complications. Trends Endocrinol Metab.

[20] Zhang H, Wang Q, He S, Wu K, Ren M, Dong H (2020). Ambient air pollution and gestational diabetes mellitus: a review of evidence from biological mechanisms to population epidemiology. Sci Total Environ.

[21] Ebert T, Stepan H, Schrey S, Kralisch S, Hindricks J, Hopf L (2014). Serum levels of irisin in gestational diabetes mellitus during pregnancy and after delivery. Cytokine.

[22] Görkem Ü, Küçükler FK, Toğrul C, Güngör T (2016). Are adipokines associated with gestational diabetes mellitus?. J Turk Ger Gynecol Assoc.

[23] Wang K, Li F, Wang C, Deng Y, Cao Z, Cui Y (2019). Serum levels of meteorin-like (Metrnl) are increased in patients with newly diagnosed type 2 diabetes mellitus and are associated with insulin resistance. Med Sci Monit.

[24] Chung HS, Hwang SY, Choi JH, Lee HJ, Kim NH, Yoo HJ (2018). Implications of circulating Meteorin-like (Metrnl) level in human subjects with type 2 diabetes. Diabetes Res Clin Pract.

[25] Lee JH, Kang YE, Kim JM, Choung S, Joung KH, Kim HJ (2018). Serum Meteorin-like protein levels decreased in patients newly diagnosed with type 2 diabetes. Diabetes Res Clin Pract.

[26] Bartha JL, Comino-Delgado R, Martinez-Del-Fresno P, Fernandez-Barrios M, Bethencourt I, Moreno-Corral L (2000). Insulin-sensitivity index and carbohydrate and lipid metabolism in gestational diabetes. J Reprod Med.

[27] Couch SC, Philipson EH, Bendel RB, Wijendran V, Lammi-Keefe CJ (1998). Maternal and cord plasma lipid and lipoprotein concentrations in women with and without gestational diabetes mellitus. Predictors of birth weight?. J Reprod Med.

[28] Lawlor DA, Ebrahim S, Davey Smith G (2004). The metabolic syndrome and coronary heart disease in older women: findings from the British Women's Heart and Health Study. Diabet Med.

[29] Li Z, Gao Z, Sun T, Zhang S, Yang S, Zheng M (2023). Meteorin-like/Metrnl, a novel secreted protein implicated in inflammation, immunology, and metabolism: a comprehensive review of preclinical and clinical studies. Front Immunol.

